# Latency-associated DNA methylation patterns among HIV-1 infected individuals with distinct disease progression courses or antiretroviral virologic response

**DOI:** 10.1038/s41598-021-02463-0

**Published:** 2021-11-26

**Authors:** Nathalia Mantovani, Alexandre Defelicibus, Israel Tojal da Silva, Maira Ferreira Cicero, Luiz Claudio Santana, Rafael Arnold, Daniela Funayama de Castro, Rodrigo Lopes Sanz Duro, Milton Yutaka Nishiyama-Jr, Inácio Loiola Meirelles Junqueira-de-Azevedo, Bosco Christiano Maciel da Silva, Alberto José da Silva Duarte, Jorge Casseb, Simone de Barros Tenore, James Hunter, Ricardo Sobhie Diaz, Shirley Cavalcante Vasconcelos Komninakis

**Affiliations:** 1 Retrovirology Laboratory, Infectious Diseases Division, Federal University of São Paulo (UNIFESP), Rua Pedro de Toledo 669, Vila Clementino, Sao Paulo, SP 04039-032 Brazil; 2 Laboratory of Bioinformatics and Computational Biology, A.C. Camargo Cancer Center, Rua Taguá, 440, São Paulo, SP 01508-010 Brazil; 3 Laboratório de Toxinologia Aplicada, Instituto Butantan, Avenida Vital Brasil, 1500, São Paulo, SP 05503-900 Brazil; 4 Laboratório de Investigação Médica 56 (LIM/56), Faculdade de Medicina FMUSP, Universidade de São Paulo, Avenida Dr. Enéas Carvalho de Aguiar, 470, São Paulo, SP 05403-000 Brazil

**Keywords:** HIV infections, DNA methylation, Viral infection, Epigenetics analysis

## Abstract

DNA methylation is one of the epigenetic modifications that configures gene transcription programs. This study describes the DNA methylation profile of HIV-infected individuals with distinct characteristics related to natural and artificial viremia control. Sheared DNA from circulating mononuclear cells was subjected to target enrichment bisulfite sequencing designed to cover CpG-rich genomic regions. Gene expression was assessed through RNA-seq. Hypermethylation in virologic responders was highly distributed closer to Transcription Start Sites (p-value = 0.03). Hyper and hypomethylation levels within TSS adjacencies varied according to disease progression status (Kruskal–Wallis, p < 0.001), and specific differentially methylated regions associated genes were identified for each group. The lower the promoter methylation, the higher the gene expression in subjects undergoing virologic failure (R = − 0.82, p = 0.00068). Among the inversely correlated genes, those supporting glycolysis and its related pathways were hypomethylated and up-regulated in virologic failures. Disease progression heterogeneity was associated with distinct DNA methylation patterns in terms of rates and distribution. Methylation was associated with the expression of genes sustaining intracellular glucose metabolism in subjects undergoing antiretroviral virologic failure. Our findings highlight that DNA methylation is associated with latency, disease progression, and fundamental cellular processes.

## Introduction

The human DNA is the object of chemical modifications, in which a methyl group is transferred to cytosine in CpG dinucleotides. This is one of the biochemical processes that make up the epigenetic information, which maintains genome integrity and plays a critical role in the configuration of transcription programs^1,2^.

Epigenetic mechanisms have been shown to sustain HIV latently infected cells^3^. The viral promoter was hypermethylated in latently infected cells in vitro^4^, indicating that methylation of the HIV promoter regulates the reactivation of the provirus. However, the long terminal repeat (LTR) in resting CD4 + lymphocytes of infected individuals was scarcely methylated^5^. Besides, the CpG methylation status of LTR and HIV-1 genes among HIV-1 infected individuals, including those who naturally control the infection, was predominantly non-methylated^6^.

Under the latent condition, the virus is silenced, and this makes cellular recognition by the immune system more difficult, thus allowing more prolonged survival of latently infected cells^7,8^. Considering the half-life of memory T CD4 + cells bearing archived provirus and the total pool of the reservoir, more than 70 years of combination antiretroviral treatment (cART) would be required to achieve a cure^9^. However**,** despite cART, some HIV infected cells are still able to produce virions that are detected through ultrasensitive methods^10^.

Expression of HIV provirus in latently infected cells has been considered a critical approach to obtain HIV eradication. The administration of latency reversal agents (LRA), such as histone deacetylase inhibitors (HDACi), has the potential to take the chromatin to a permissive state and to activate virus transcription^8,11,12^. However, purging strategies have failed to reduce the size of the HIV reservoir^13,14^, possibly because of the low immunological functions of the infected patients^15,16^. Hence, a better understanding of HIV-1 persistence and its underlying features is critical for developing new therapeutic strategies to achieve HIV remission without antiretrovirals.

Although HIV-1 infection was associated with DNA methylation changes of cellular genes^17–19^, we noticed a lack of studies addressing CpG-rich genome regions' methylation status through a large scale approach to investigate patterns potentially associated with latency. Here, we hypothesized that latent infection and heterogeneous courses of HIV infection are subject to different DNA methylation patterns in the human genome. Additionally, our data indicated that CpG methylation is one of the underlying mechanisms shaping gene expression and pathways involved in regulating intracellular glucose metabolism in subjects experiencing cART failure.

## Results

Methylation analysis was conducted for samples of 6 controls and 22 People Living with HIV (PLWH). Out of the total number of PLWH analyzed, seven were cART virologic responders, seven were cART virologic failures, six long-term nonprogressors (LTNP), and two elite controllers (EC). The Class I HLA alleles for the elite controllers and for four out of the six long term non-progressors are shown in Supplementary Table 1. Among the control group, three were males, and three were females (mean age of 31 years), whereas among HIV-1 infected individuals, 12 were males and 10 were females (mean age of 43 years).

The mean time since HIV diagnosis for all HIV infected individuals was 13 years (3–22 years). In the virologic responders' group, the mean duration of cART was six years, and the median CD4^+^ count was 555 cells/mm^3^ (118–985 cells/mm^3^). In the virological failure group, the mean duration of cART was eight years, the median CD4^+^ count was 240 cells/mm^3^ (159–623 cells/mm^3^), and the median viral load was 3.66 log_10_ (1.8–4.57 log). In the group of LTNP, the median CD4^+^ T cell count was 693 cells/mm^3^ (566–1,257 cells/mm^3^), and the median viral load was 3.40 log_10_ (2.3–4.04 log). EC exhibited a median T CD4^+^ count of 1401 cells/mm^3^ (1,190–1,613 cells/mm^3^). A detailed characterization of the HIV infected individuals enrolled in the study is depicted in Table 1.

**Table 1:** Demographic, clinical and laboratory characteristics of the analyzed individuals.

ID	Gender	Age (Years)	CD4 (cells/mm^3^)	Viral load (log 10 copies/mL)	Year of diagnosis	Year of sampling	Year of HAART start	HAART scheme
CT2	M	34	–	–	–	2015	–	–
CT3	F	30	–	–	–	2015	–	–
CT4	F	28	–	–	–	2015	–	–
CT5	F	32	–	–	–	2015	–	–
CT6	M	32	–	–	–	2015	–	–
CT7	M	30	–	–	–	2015	–	–
LTNP_1	M	41	566	4.04	1994	2012	–	–
LTNP_2	M	32	848	3.18	2006	2012	–	–
LTNP_3	M	51	681	3.53	1999	2013	–	–
LTNP_4	F	55	706	3.28	1993	2014	–	–
LTNP_5	F	29	1257	2.3	2010	2014	–	–
LTNP_6	M	49	582	3.55	1991	2012	–	–
EC_1	F	42	1613	< 40	2003	2013	–	–
EC_2	F	54	1190	< 40	1996	2013	–	–
VR_1	M	42	600	< 40	1998	2014	2008	3TC + ZDV + EFV
VR_2	M	44	501	< 40	2003	2014	2003	3TC + ZDV + TDF + LPV/r
VR_3	F	36	555	< 40	1996	2013	2013	3TC + TDF + DRV/r
VR_4	M	49	985	< 40	1993	2014	1997	3TC + ZDV + LPV/r
VR_5	M	41	486	< 40	1999	2014	2013	3TC + TDF + DRV/r + RAL
VR_6	M	41	118	< 40	2004	2014	2005	TDF + ABV + ATV/r
VR_7	F	40	602	< 40	2006	2014	2009	3TC + TDF + ATV/r
VF_1	M	50	228	4.57	2010	2014	2010	3TC + EFV + TDF
VF_2	F	40	159	4	1997	2014	2009	3TC + ZDV + DRV/r
VF_3	F	53	232	3.33	2000	2014	2002	3TC + ABV + ATV
VF_4	F	53	260	4.23	2000	2014	2000	3TC + ZDV + LPV/r
VF_5	M	35	623	1.91	2009	2014	2002	3TC + ZDV + ATV/r
VF_6	F	41	406	2.08	1997	2014	1998	3TC + ZDV + TDF + DRV/r
VF_7	M	44	473	1.8	1998	2014	2012	3TC + TDF + ATV/r

*3TC* Lamivudine, *ABV* Abacavir, *ATV* Atazanavir, *ATV/r* Atazanavir + Ritonavir, *CT* Control, *DRV* Darunavir, *DRV/r* Darunavir + Ritonavir, *EFV* Efavirenz, *E.C.* Elite controller, *F* Female, *HAART* Highly active antiretroviral therapy, *LPV/r* Lopinavir + Ritonavir, *LTNP* Long-term non-progressor, *M* Male, *RAL* Raltegravir, *TDF* Tenofovir, *V.F.* Virologic Failure, *V.R.* Virologic Responder, *ZDV* Zidovudine.

There were no statistically significant differences in sex (p-value > 0.05) and time since HIV diagnosis (p-value > 0.05). The groups significantly differ in age (p-value = 0.015) and CD4 T cells counts (p-value = 0.007). However, according to the deconvolution analysis, modest effects on CD4 T cells fractions were observed (Suplementary Table 2, Supplementary Fig. 1). Each sample achieved an average of 65 million paired-end alignments with a unique best hit (see Supplementary Table 3), yielding billions of CpGs with sodium bisulfite conversion rate of 98.9% (see Supplementary Table 3) designed to cover CpG islands, shores (up to 2 kb flanking CpG islands), shelves (up to 2 kb flanking shores), promoters, RefGenes and cancer-specific sites. Read depth distribution after filtration is depicted in Supplementary Fig. 2. As expected for differentiated cells, CpG methylation levels followed a bimodal fashion (see Supplementary Fig. 3), which is set up during embryogenesis^20^.

### Disease progression heterogeneity was associated with different DNA methylation patterns

**Figure 1:**
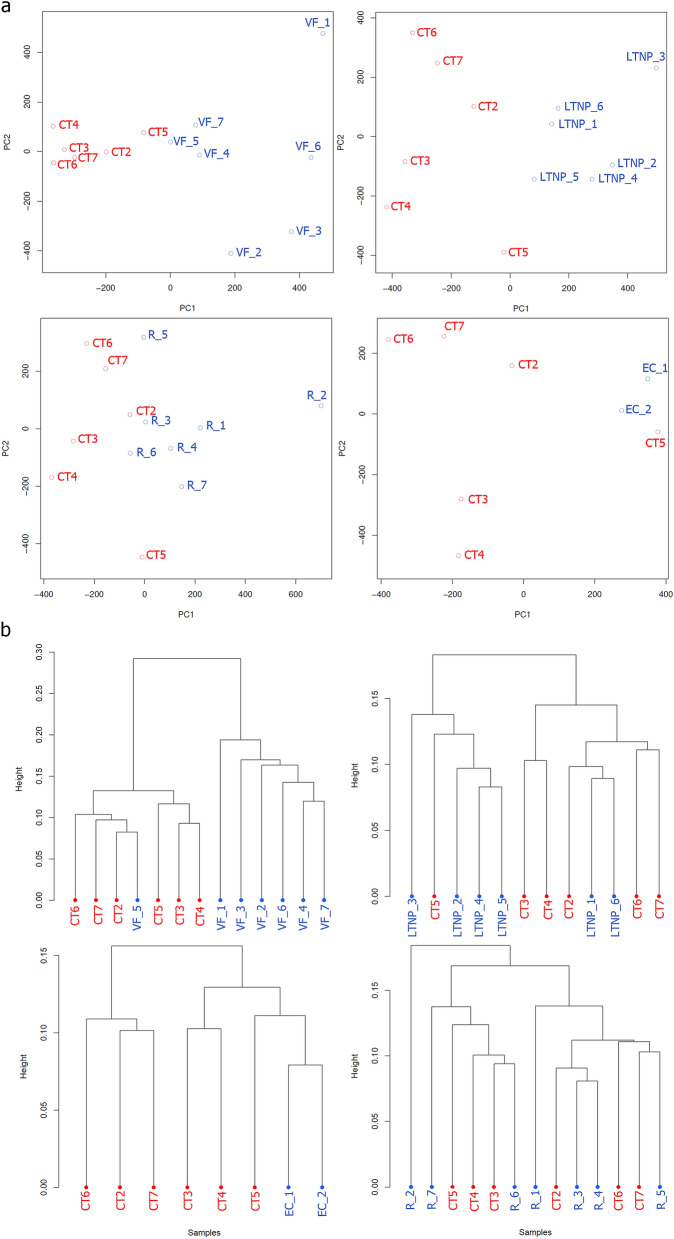
Clustering analysis of CpG methylation profiles. (**a**) Principal Component Analysis (PCA) of four methylation profiles comparisons between HIV groups and controls. (**b**) Hierarchical clustering of four methylation profiles comparisons based on 1-Pearson’s correlation distance. Red denotes healthy controls and blue denotes HIV samples. *CT* Control group, *EC* Elite controller, *LTNP* Long-term nonprogressor, *R* virologic responder, *VF* virologic failure.

Methylation profiles between each HIV infected group and controls are represented by Principal Component Analysis (PCA) (Fig. 1a) and hierarchical clustering analysis using the correlation distance method (Fig. 1b). Samples grouped closer are similar in their methylation profiles. Pearson’s correlation scores are depicted in Supplementary Fig. 4.

Each HIV infected group was compared to control group in order to identify significant differentially methylated regions (DMR). The highest number of DMR was detected in virologic failures, whereas the virologic responders exhibited the lowest number. Out of the 11,299 DMR, virologic failures and EC accounted for 4,733 and 3,600 regions, respectively; LTNP and virologic responders accounted for 1,965 and 1,001 regions, respectively (see Supplementary Table 4).

**Figure 2:**
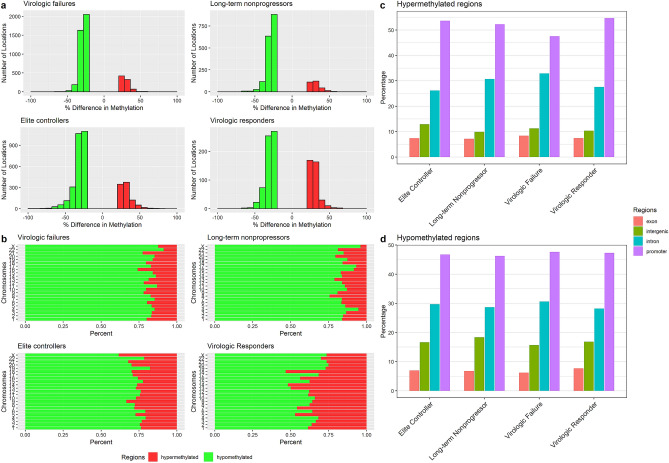
Differential methylation analysis. Methylation percentages were calculated for windows encompassing 100 bp. Then, methylation percentages for each region in HIV groups were compared against a control group. A cutoff of ≥ 25% for methylation difference and a q-value of < 0.01 were considered for the analysis. (**a**) Number of locations showing hyper and hypomethylation in HIV groups compared with controls. (**b**) Percent of hypo and hypermethylated regions across human chromosomes. (**c**) Annotation of differential methylation showing the percentage of differentially hypermethylated regions distributed across exons, intergenic, introns and promoter regions. (**d**) Annotation of differential methylation showing the percentage of differentially hypomethylated regions distributed across exons, intergenic, introns and promoter regions.

Concerning the methylation status and genomic location of DMR, we were able to identify a higher number of hypomethylated than hypermethylated sites in all HIV-1 groups (Fig. 2a), widely distributed across somatic and X human chromosomes (Fig. 2b), and nearly half colocalized with gene promoters computed from 1 kb upstream to 1 kb downstream of Transcription Start Sites (TSS) (Fig. 2c,d). Out of the DMR that colocalized with promoters in each group, 83.4% (n = 772) was hypomethylated in LTNP, followed by 82.6% (n = 1860) in subjects undergoing cART failure, EC with 71.7% (n = 1,250), and virologic responders with 58.8% (n = 294).

**Figure 3:**
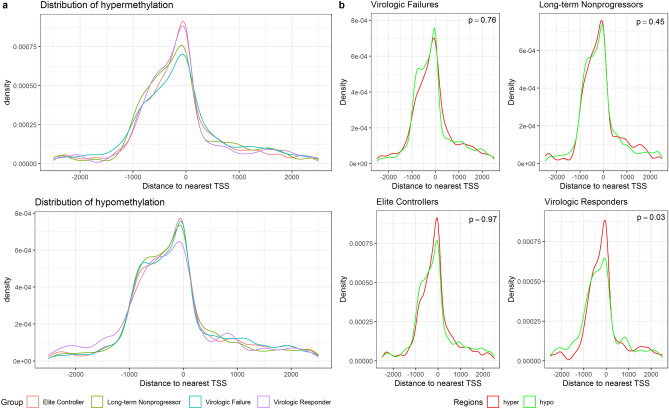
Distance from differentially methylated regions to nearest TSS in base pairs. (**a**) Distribution of hypo and hypermethylated regions in HIV-infected groups. (**b**) Intragroup comparison of hypo and hypermethylation distributions surrounding TSS for each HIV group (Wilcoxon rank-sum test). *TSS* Transcription Start Site.

Figure 3a represents hypo and hypermethylation distributions among HIV groups. Although intragroup comparisons of DMR distributions revealed slightly distinct patterns depending on the status of disease progression, only virologic responders exhibited higher distribution of hypermethylated regions closer to TSS (Wilcoxon rank-sum test, p-value = 0.03, Fig. 3b).

**Figure 4:**
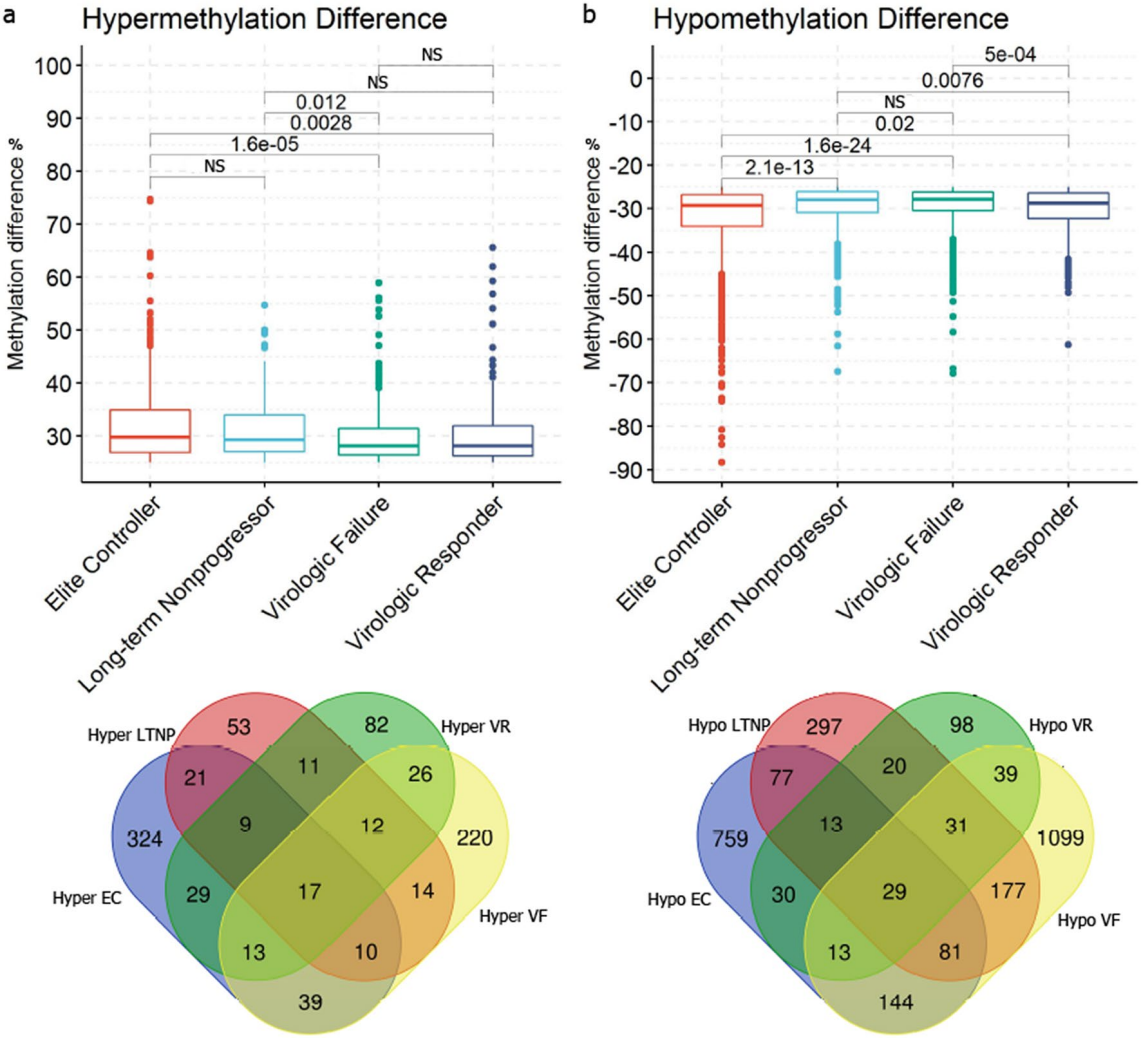
Differentially methylated regions within − 1 kb/ + 1 kb surrounding Transcription Start Sites. Significant regions (≥ 25% for methylation difference and q-value < 0.01) were annotated to find DMR-associated genes. DMR within − 1 kb/ + 1 kb flanking TSS was filtered for comparing hypo and hypermethylation differences. (**a**,**b**) Boxplots show comparisons of percentages for significant hypermethylated and hypomethylated promoters among HIV groups (Kruskal–Wallis rank-sum test < 0.001). P-values were calculated using the Wilcoxon rank-sum test with Benjamim-Hochberg correction for multiple comparisons and non-significant values were omitted. Venn-diagrams represent the number of DMR-associated genes detected for each group and their intersections. *NS* Non-significant p-values.

Hypo and hypermethylation medians within − 1 kb/ + 1 kb flanking TSS were significantly different among HIV groups. EC showed the highest median of hypermethylation (29.73%), followed by LTNP (29.28%), virologic responder (28.11%), and the lowest was exhibited by virologic failures (28.09%) (Kruskal–Wallis, p < 0.001) (Fig. 4a). Interestingly, hypermethylation levels between groups that spontaneously control HIV infection were not statistically different (p > 0.05); similarly, hypermethylation levels between HIV groups that do not have such ability were not significantly different as well (p > 0.05) regardless of the plasma viral load. Moreover, hypermethylation levels from LTNP were significantly higher than virologic failures (p = 0.012) irrespective of the similar median viral loads (3.66 log_10_ for virologic failures and 3.40 log_10_ for LTNP), providing further support for an association between DNA methylation and natural control of HIV infection. Concerning hypomethylation, the highest level of hypomethylation was observed for EC (− 29.29%), followed by virologic responders (− 28.72%), LTNP (− 27.95%), and virologic failures (− 27.82%) (Kruskal–Wallis, p < 0.001) (Fig. 4b).

It is worth mentioning that we were able to detect a methylation signature previously identified in PLWH compared to HIV uninfected subjects^21^. The promoter of the NLR family, CARD domain-containing gene 5 (NLRC5), involved in activating major histocompatibility complex class I gene expression^22^, was hypomethylated for all HIV groups except EC (see Supplementary Table 4). Out of the genes that were hypomethylated except for EC, changes in methylation for the individual groups were significant only for the region chr8:27397101–27397200, which corresponds to the Protein Tyrosine Kinase 2 Beta (PTK2B) (Supplementary Fig. 5). We also identified other DNA methylation signatures previously associated with low and high plasma viral loads^23^. For instance, promoters of RUNX family transcription factor 3 (RUNX3), Deltex E3 Ubiquitin Ligase 3L (DTX3L), and Interferon Induced Protein 44 Like (IFI44L) were hypomethylated only in virologic failures; MX Dynamin Like GTPase 1 (MX1) promoter exhibited higher hypomethylation level for virologic failures than for virologic responders, and Lymphocyte Cytosolic Protein 2 (LCP2) promoter was hypomethylated in EC and hypermethylated in virologic responders (see Supplementary Table 4).

### DNA methylation patterns were associated with distinct biologic pathways in HIV infected groups

DMR-associated biologic pathways in our data included immune response, cell signaling, and metabolic regulation (see Supplementary Table 5). Hypermethylation in virologic failures was associated with T cell receptor signaling pathway, including CD4 gene, whose promoter was hypermethylated; hypomethylation in such patients was associated with TNF-signaling, Phosphatidylinositol-3-kinase/Protein Kinase B (PI3K/Akt) signaling pathway, Hypoxia-inducible factor 1 (HIF-1), among other pathways. Conversely, HIF-1 signaling was enriched for hypermethylated regions in LTNP. This pathway coordinates the transcription activation of several genes involved in glucose metabolism, angiogenesis, and cell survival^24,25^. According to our data, provided that the HIF-1 pathway was related to hypermethylation in subjects that naturally sustain viral loads to low levels and hypomethylation in individuals experiencing cART failure, methylation was a plausible aid to configure the expression of genes involved in regulating glycolysis and its interdependent cascades.

### DNA methylation inversely correlated with gene expression in subjects experiencing virologic failure

**Figure 5:**
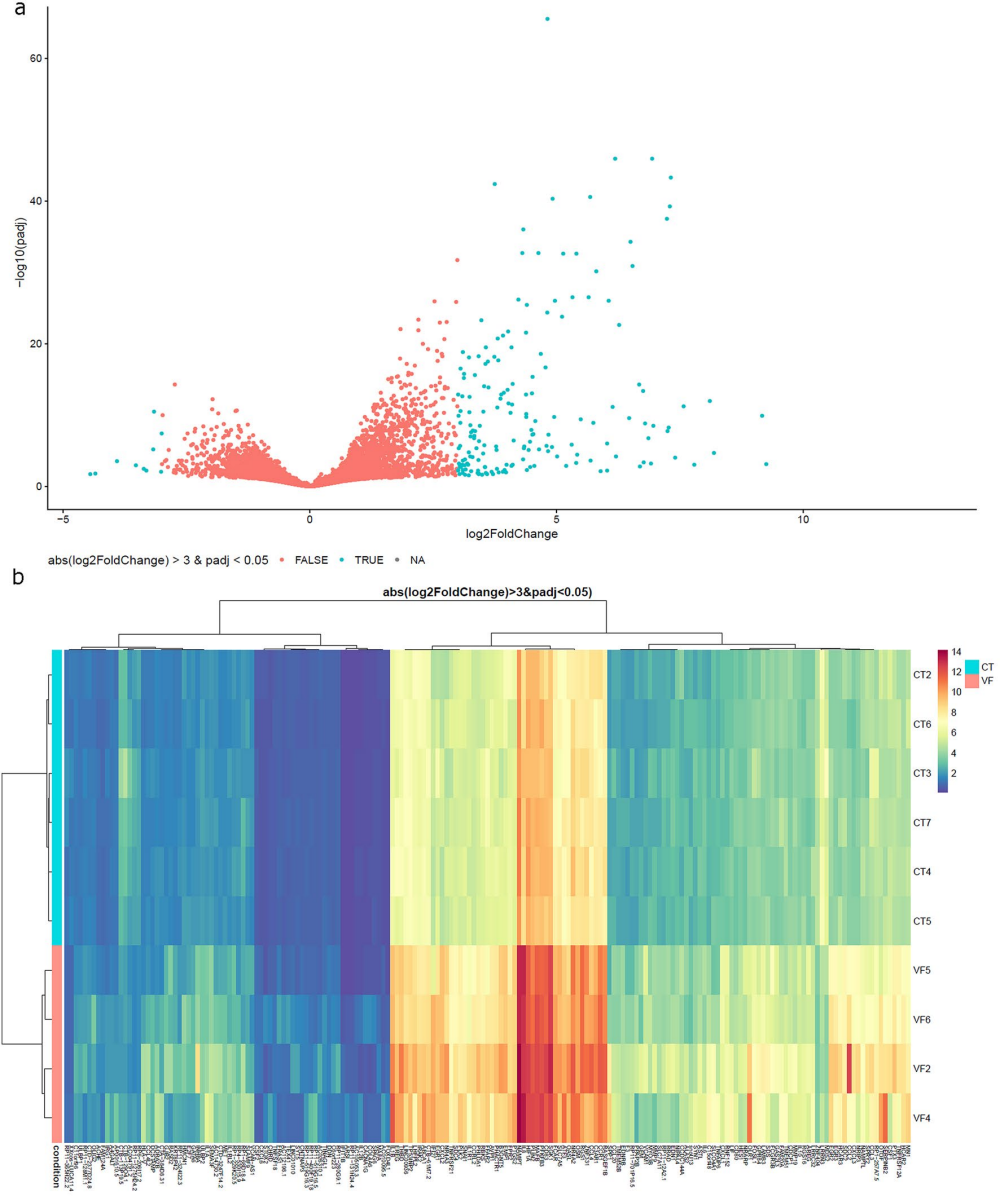
Gene expression analysis. RNA-seq data of virologic failures were compared to control group. (**a**) Volcano plot reports p-adjusted in the y-axis against the fold change of gene expression in the x-axis. Blue denotes differentially expressed genes considering log2 fold change > 3 and p-adjusted < 0.05. Positive and negative values for log2 fold change indicate up-regulated and downregulated genes, respectively. (**b**) Heatmap illustrates differential expression data for six controls and four virologic failures. Blue and red indicate lower and higher transcription levels, respectively. *CT* Control group, *VF* Virologic failure.

To better understand the interplay between methylation and gene expression in “Patients” experiencing virologic failure, RNA-seq analysis was conducted for six controls and four virologic failures. We were able to find 187 differentially expressed genes (DEG), considering log2 fold change > 3 and p-adjusted < 0.05 (see Supplementary Table 6). The saturation curve for each sample is shown in Supplementary Fig. S6. The observation that most genes were up-regulated in virologic failures (Fig. 5a,b) is consistent with the higher number of hypomethylated regions found in this group (Fig. 2a,b). Biological pathways for RNA-seq data are depicted in Supplementary Table 7 Some of the up-regulated genes were Interleukin-6 (IL-6) and Interleukin-1 beta (IL-1β), two well known pro-inflammatory mediators^26,27^, and also Interleukin-10. Previous reports demonstrated that the natural suppression of HIV was associated with the highly functional co-expression of cytokines such as TNF and IL-10 concomitant with significant maintenance of anti-inflammatory and anticoagulant properties^28^.

**Figure 6:**
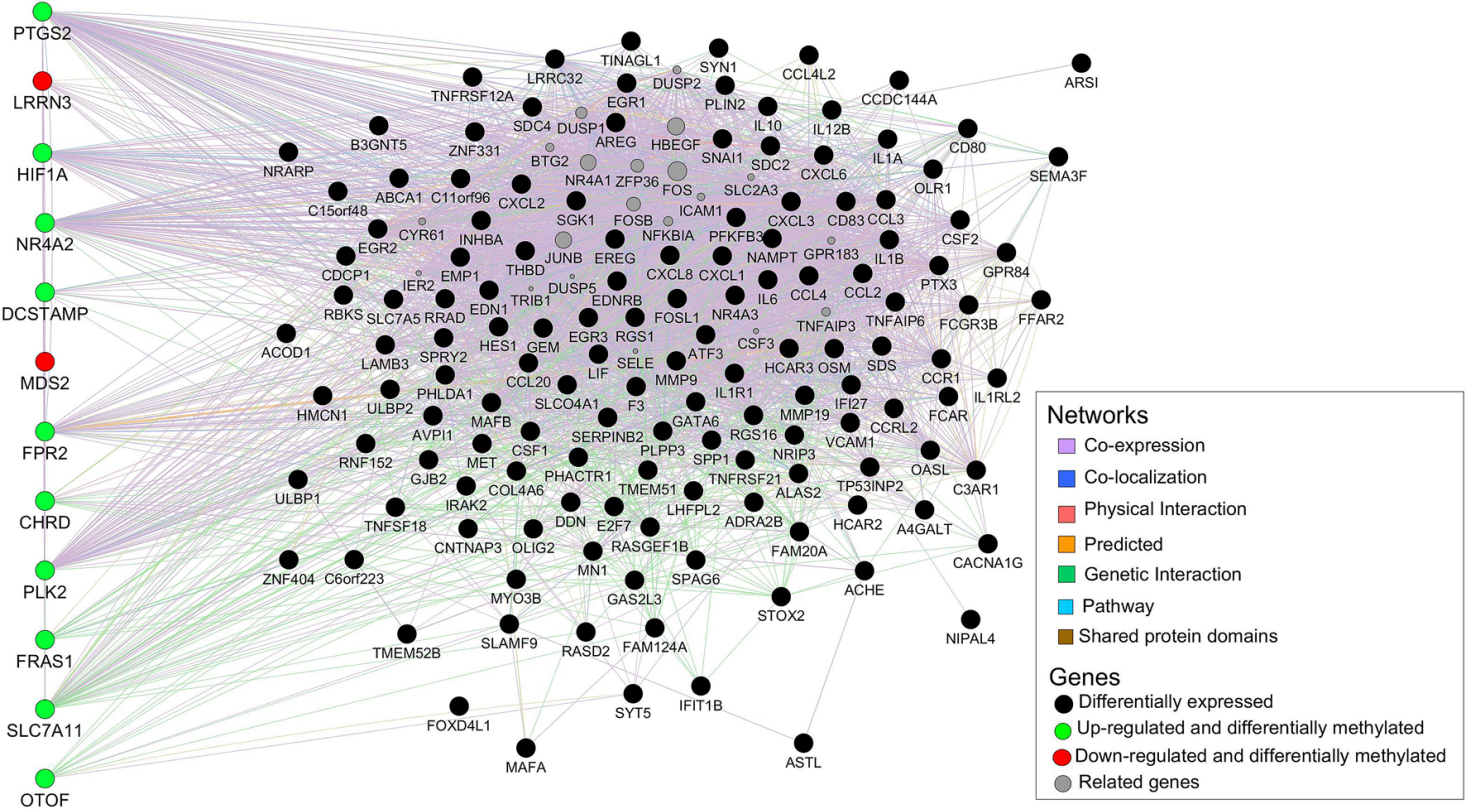
Network for differentially expressed genes in individuals failing cART. Different types of network were identified among the 187 transcripts associated with virologic failure. Genes with methylation and gene expression correlation are showed in the left. HLA-V is a pseudogene and was not represented.

**Figure 7:**
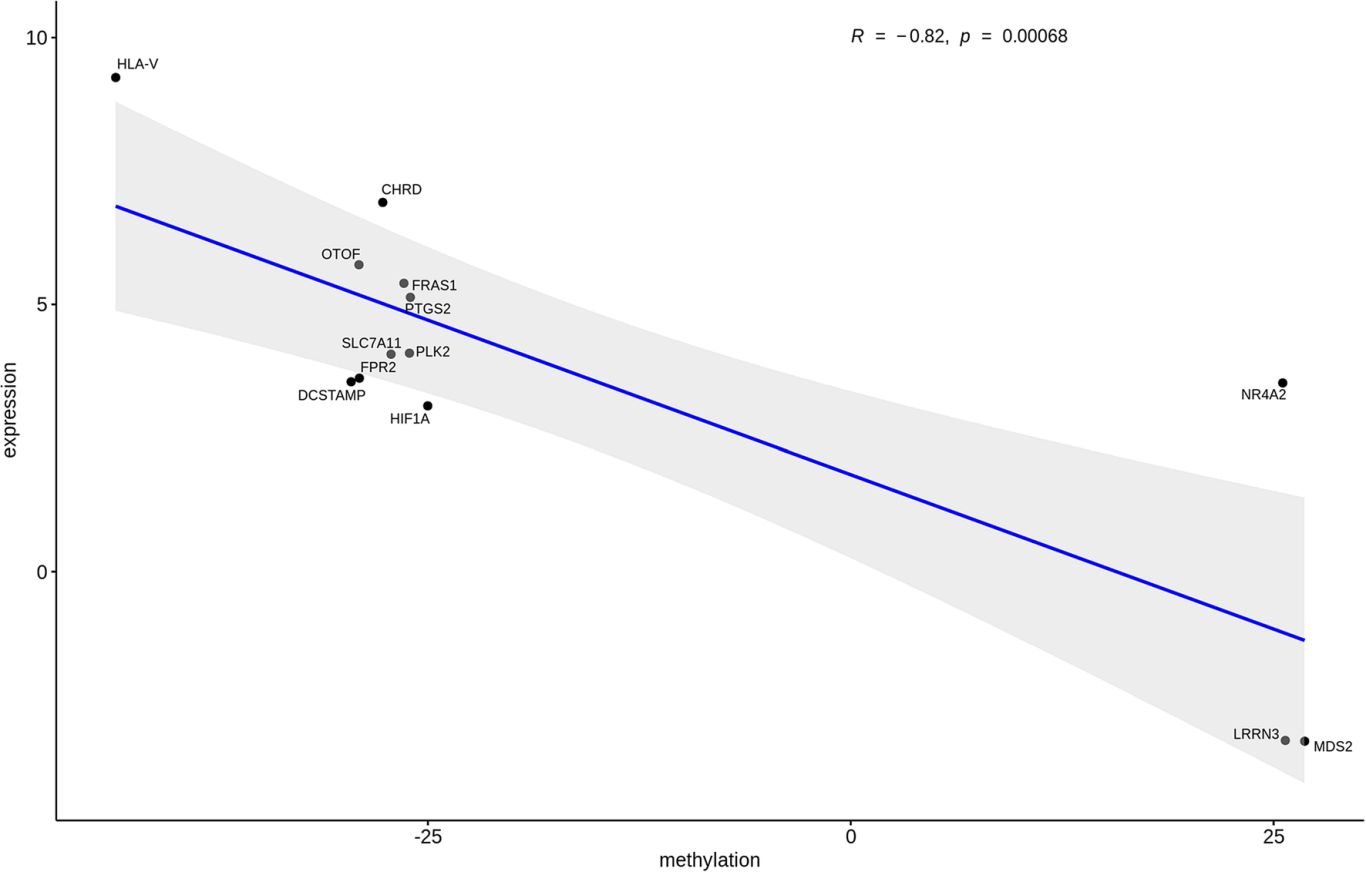
Gene expression and methylation correlation. Pearson’s correlation coefficient of the subset of DEG genes with their corresponding differentially methylated promoter. For genes having more than one DMR (FRAS1 and MDS2), the mean difference in methylation was calculated and considered for the correlation analysis. Confidence intervals are shown in grey shading. *DEG* Differentially expressed genes, *DMR* Differentially methylated regions.

Out of the 187 DEG genes, 13 showed differentially methylated promoters (Table 2) and established different types of networks (Fig. 6). Promoter methylation and gene expression were inversely correlated in subjects experiencing cART failure (R = − 0.82, p = 0.00068) (Fig. 7). DNA methylation and gene expression negatively correlated in our data corroborate the long-term conception that promoters highly methylated associates with transcriptional silencing^29–31^.

**Table 2:** Subset of genes with DNA methylation and gene expression association in subjects failing cART compared to control group.

Gene	Methylation difference	q-value (Methylation difference)	Distance to TSS	log2 FoldChange	p-adj (log2 foldchange)
MDS2*	27.52	7.78e−19	−34	−3.18	6.54e−06
MDS2*	26.15	9.61e−12	−234	−3.18	6.54e−06
LRRN3	25.69	1.66e−51	9	−3.16	3.71e−11
HIF1A	−25.01	2.34e−23	308	3.10	1.64e−19
NR4A2	25.54	1.67e−169	522	3.53	1.01e−14
DCSTAMP	−29.54	1.28e−51	−87	3.55	2.3e−03
FPR2	−29.05	7.69e−13	643	3.62	1.59e−14
SLC7A11	−27.18	2.77e−14	−553	4.07	5.13e−11
PLK2	−26.09	4.15e−21	195	4.09	3.35e−20
PTGS2	−26.04	3.41e−35	−133	5.13	2.47e−33
FRAS1*	−26.99	4.39e−11	−138	5.40	5.16e−04
FRAS1*	−25.85	1.98e−15	−731	5.40	5.16e−04
OTOF	−29.08	2.33e−26	−604	5.74	2.33e−26
CHRD	−27.67	5.21e−09	115	6.91	6.22e−04
HLA−V	−43.46	3.16e−67	349	9.25	7.49e−04

*CT* Control group, *VF* Virologic failure group, *TSS* Transcription Start Site.

*Differentially expressed genes associated with two differentially methylated regions.

Hypoxia-inducible factor 1-alpha (HIF-1α) was up-regulated and hypomethylated in virologic failures. This is consistent with the HIF-1 signaling pathway being one of the most significant pathways enriched for hypomethylated regions in “Patients” failing cART. HIF-1α is a subunit of the HIF-1 transcription factor primarily activated in response to microenvironment oxygen deprivation^24,32^, although oxygen-independent regulation may increase its expression in T cells^33^. Inside the nucleus, HIF-1 attaches to Hypoxia Response Element within regulatory sequences of target genes, rendering the cells adapted to survive under low oxygen supply^24,25,33^.

Finally, although analyses were conducted in Peripheral Blood Mononuclear Cells (PBMC), which consists of a large pool of mononuclear cell subpopulations, certain alterations are likely associated with specific cell subsets. Nuclear Receptor Subfamily 4 Group A, Member 2, (NR4A2), which codes for a steroid/thyroid hormone nuclear receptor^34^, was the only up-regulated gene with a hypermethylated promoter, which is in agreement with a previous report^35^. NR4A2 plays a regulatory role in the development of dopaminergic neurons^36^ and, interestingly, monocytes from HIV-1 patients with cognitive impairment showed the same epigenetic and expression profile for NR4A2^35^.

## Discussion

The current study aimed at charting the methylation landscape of HIV-1 infected individuals heterogeneous in terms of HIV viremia control. We evaluated a panel of samples comprising individuals that naturally control HIV viremia (EC), partially naturally control the HIV harm to the immunity (LTNP), individuals that artificially control HIV viremia (virological responders), and individuals that were not able to artificially suppress HIV replication (virologic failures) Our major findings revealed distinct methylation patterns among PLWH distinct groups, concerning distribution and levels of methylation within human genome segments associated with transcription regulation. Additionally, we were able to associate promoter methylation with changes in gene expression for virologic failures, providing further insights into the role of epigenetic machinery on HIV persistence.

Cytosines followed by guanines (5’-CpG-3’) in the human genome are methylated in a reaction catalyzed by DNA methyltransferases, which were shown to be increased upon HIV infection in vitro^17,19,37,38^. Furthermore, global DNA methylation percentage was higher in HIV infected CD4 + T cells in vitro^39^*,* and overall DNA hypermethylation was associated with HIV infection in a pair of serodifferent monozygotic twins^19^. However, a different dynamic was observed in our clinical samples, which is in agreement with a previous study addressing the DNA methylation status of a large cohort of HIV-infected and non-infected subjects^21^ that showed a higher number of hypomethylated regions in PLWH.

Unexpectedly, nearly half of the disturbed regions colocalized with promoters, indicating a potential to affect gene expression^1,40^. Roughly 70% of promoters locate within CpG islands (CGI)^41^ and the experiment is aimed at addressing CpG-dense segments. However, intergenic segments constitute 75% of the human genome, and introns account for nearly 24%^42^. Additionally, CpG sites spread throughout the genome are highly methylated, whereas CGI-associated promoters are markedly non-methylated^1,43^, then it is counter-intuitive that DMR was promoter-enriched and mostly hypomethylated. Given that HIV integrates into the human genome and harnesses the host transcription factors to complete its replication cycle, our data suggest that HIV-infected cells are primarily hypomethylated, which might favor the replenishment of infectious particles in the blood circulation through efficient provirus transcription. Nevertheless, considering that only a minority of cells in the bulk of PBMC is HIV infected^44,45^, we cannot provide clues on the exact mechanism by which the DNA is undergoing demethylation in such patients.

Individuals on fully suppressive cART exhibited higher hypermethylation distribution closer to TSS. Conversely, hypo and hypermethylation distribution surrounding TSS were similar in subjects whose viral loads were detectable. This slightly different distribution may reflect the contingent of cells developing active replication and their latently infected counterparts. cART targets virus-producing cells, then a relatively greater amount of latently infected cells may be represented in individuals on a successful cART contributing to the higher hypermethylation closer to TSS. This idea is in line with longitudinal analysis showing that cART negatively selected single provirus integration in host genes expressed at higher rates^46^, suggesting that provirus integrated within poorly expressed regions are more prone to perpetuate and become part of the archived provirus. Indeed, pharmacological agents aiming at forcing expression of HIV-1, such as HDACi, efficiently stimulated viral transcription from latently infected cells and turned chromatin more permissive for HIV expression by increasing global acetylation^13,14,47^. However, to date, “kick and kill” strategies have not successfully reduced viral reservoir, even when HIV-specific T cell response was boosted with viral vector vaccine^48^, indicating that additional approaches might be necessary to “kill” infected cells reactivated from latency. On the other hand, if DNA methylation is regarded as a relatively stable epigenetic modification in mammalian genomes^49^, and hypermethylation surrounding TSS appears to be associated with latently infected cells, our data reinforce findings from an in vitro proof-of-concept study highlighting that the combination of multiple LRA should be considered to activate the overall contingent of cells refractory to HIV expression^50^.

The observation that disease progression and/or viral control heterogeneity was subject to distinct hypermethylation levels suggests that DNA methylation is one of the underlying mechanisms associated with in vivo HIV control. Recently, specific DNA methylation signatures were associated with innate and adaptive immune responses in untreated individuals with high and low viral^23^. DTX3L, IFI44L, and MX1 were part of a cluster containing hypomethylated genes involved in antiviral response in HIV infected subjects with high viral loads, whereas LCP2 was part of a hypermethylated cluster associated with adaptive response in such individuals^23^. To broaden this understanding, our data demonstrated that individuals who naturally control HIV infection exhibited higher hypermethylation rates within TSS adjacencies, perhaps promoting an autologous block and lock phenomena. Conversely, lower rates of hypermethylation were observed for subjects unable to naturally control HIV viremia, irrespective of viral loads. Furthermore, hypermethylation levels were higher for EC compared to virologic responders, suggesting the importance of this phenomenon in the natural control of viremia. Collectively, our data provide strong evidence that DNA hypermethylation distribution associates with the contingent of latently infected cells, and high hypermethylation rates associates with spontaneous control of HIV infection. It remains to be elucidated mechanisms that make the host naturally control the HIV viremia by epigenetic processes such as DNA methylation or the genetic correlates for HIV viral control. For instance, a deep metabolomic evaluation found evidence that EC harbors an inborn error of metabolism (late-onset multiple acyl-coenzyme A dehydrogenase deficiency [MADD])^51^.

Regarding the DMR-associated biologic pathways, HIF-1 signaling was enriched for hypermethylated regions in LTNP, while it was associated with hypomethylation in the virologic failure group. The latter also showed hypomethylation and up-regulation of HIF-1α, a subunit of the transcription factor HIF-1, whose activity is triggered by intracellular hypoxia^24,52^. However, hypoxic conditions inhibited HIV activation in vitro*,* with cells maintaining stable activation and viability^53^. Since patients experiencing virologic failure exhibit productive infection, it is conceivable that the increased expression of HIF-1α was driven by factors other than intracellular hypoxia. Indeed, HIF-1α may be triggered in normoxic T cells by Vpr-induced oxidative stress^54^ and mitochondrial Reactive Oxygen Species (ROS)^55^. Mitochondrial ROS produced in response to cytosolic dsDNA induced HIF-1α expression in HIV infected cells irrespective of viral accessory proteins^55^. Upon activation of naïve T cells, metabolic reprogramming from oxidative phosphorylation to aerobic glycolysis takes place for potent T cell effector function and cytokine production^56,57^, which is supported by HIF-1α through upregulation of glucose transporter-1 (GLUT-1)^33,58^. T cells cultured in glycolysis deprivation fail to secrete Interferon-γ through a mechanism that prevents its translation^57^. By contrast, HIV infection also relies on cellular bioenergy status, evidenced by a reduction of HIV particle assembly when glucose metabolism was blocked in T CD4 cells^59^. Moreover, CD4 T cells and monocytes of HIV-infected patients were associated with increased phenotype for glucose metabolism irrespective of cART^60,61^.

Glycolysis is not regulated only by HIF-1 signaling. Along with the increased expression of HIF-1α accompanied by HIF-1 pathway markedly associated with hypomethylation, we observed that hypomethylated regions were also enriched for PI3K-Akt signaling in individuals undergoing virologic failure. Upon activation of AKT by a PI3K-dependent signaling, downstream cascades control a variety of elementary processes, which include glucose metabolism by increasing the activity of GLUT-1^62,63^, bringing further support for the idea that methylation is associated with transcription of genes implicated on intracellular glucose metabolism in such individuals. However, further experiments are necessary to evaluate causality between methylation of individual regions and biologic pathways.

Some limitations in the present study must be emphasized. We recognize the absence of data related to the time of infection of each patient to correlate with differences in methylation. Although the results suggested that DNA methylation was associated with latently infected cells, our experiment was not designed to exclusively characterize latently HIV infected cells and does not allow the discrimination among latently infected cells, virus producing cells and uninfected bystander cells. While we found no statistic significance between hypermethylation levels within the gene promoters of virologic failures and responders groups, suggesting that similar hypermethylation rates between such groups might be associated with the viral rebound that responders would experience upon treatment interruption, we recognize that it is challenging to speculate the mechanistic role played by methylation alterations of individual genes in the viral rebound. Gene expression and methylation were inversely correlated, and distinct phenotypes for disease progression were associated with specific methylation patterns. However, it is not possible to establish causality, as methylation may reinforce transcriptional silence that was consolidated by other epigenetic modifications rather than cause the shutdown of gene expression^64^. Deconvolution analysis showed that the differences in CD4^+^ T cell fractions were not statistically significant. However, the hypermethylation levels follow the same trend of CD4^+^ T cell counts. The presence of multiple cellular subpopulations might influence the methylation variability observed in epigenetic studies^65^, once the PBMC are constituted by cellular lineages with differences towards their methylation patterns^66^. Although the groups significantly differ in age, a previous report that developed an epigenetic clock to estimate the DNA methylation age of tissues and cells subsets showed that age causes minor effects on beta values of individual CpG sites^67^. Furthermore, our analyses were restricted to circulating cells and were based on a small sample size, particularly for the EC group, which could prevent some DMRs from reaching statistical significance. Future studies addressing whether and how the methylome is disrupted in other compartments that support HIV replication may deepen the knowledge regarding the impact of epigenetic modifications on HIV infection.

Nonetheless, we have shown that DNA methylation was associated with latently infected cells and natural control of HIV infection. Furthermore, we demonstrated the association between DNA methylation and expression of genes and cascades sustaining intracellular glucose metabolism in individuals undergoing virologic failure. Our findings highlight the dynamic nature of the methylation landscape in HIV infection, which may impact future studies aiming at HIV remission without the use of antiretrovirals.

## Methods

### Patients

Clinical and epidemiological data of HIV-1-infected subjects from the outpatients' Clinics of the Federal University of São Paulo were analyzed, and candidates were selected from 2013 and 2014. Patients were asked about the possibility of taking part in the study voluntarily, and all the individuals enrolled in the study agreed and signed an informed consent term. The research was approved by the ethics committee from the Federal University of São Paulo (#51854). The methods were carried out according to the approved guidelines.

Patients were divided into (i) virologic responders group, in which viral loads were below detection limits during the preceding six months using cART; (ii) virologic failure group, consisting of detectable viral loads despite cART during the last six months; (iii) LTNP, antiretroviral naïve individuals presenting low detectable viral loads and stable CD4^+^ T cell counts above 500 cels/mm^3^ for at least three years; (iv) EC, presenting undetectable viral loads and T CD4^+^ counts above 500 cels/mm^3^ for at least seven years without cART. The control group consisted of HIV-1/HIV2 seronegative individuals, which were also negative for Hepatitis B, Hepatitis C, Chagas' disease, Syphilis, Human T Lymphotropic virus-1 (HTLV-1), and Human T Lymphotropic virus-2 (HTLV-2).

CD4 + T cell counts, and viral loads were performed with the same samples used to perform Methyl-seq and RNA-seq. Viral load was assessed using the RT-PCR *HIV-1 Abbott Real Time* assay *(*Abbott Molecular Inc*.)*, with a limit of detection of 40 copies/mL.

### Purification of DNA and RNA

PBMCs were isolated with Ficoll Paque (*GE Healthcare Life Sciences)* and stored in liquid nitrogen undisturbed until nucleic acid purification. DNA purification was performed using the *QIAamp DNA BLOOD MINI KIT (Qiagen, Valencia, CA),* according to the manufacturer's instruction. For the RNA purification, the *QIAamp* RNeasy Mini kit (*Qiagen, Valencia, CA, USA*) was used, according to the manufacturer's instructions.

### Methyl-seq library preparation

Methyl-seq was performed using the *SureSelect Methyl-seq Target Enrichment System for Illumina Multiplexed Sequencing kit (Agilent Technologies),* according to the manufacturer's instruction. Briefly, 3 µg of genomic DNA was sheared by sonication in a Covaris S2 *(Covaris, Inc)*, and the extremities of the sequences were repaired, ligated to adapters, and hybridized with biotinylated probes designed to capture CpG-rich regions of the human genome. The libraries were treated with sodium bisulfite followed by polymerase chain reaction (PCR) with primers complementary to the adapters. DNA treatment with bisulfite converts non-methylated cytosines into uracyls and, during the PCR, uracyls were amplified in the complementary strands as adenine and then as thymine. Finally, different indexes were added to each sample for them to be sequenced in a multiplex.

Libraries were validated before sequencing using the *Kapa Library Quantification* kit *(Kapa Biosystems*)*,* and the size of the fragments was analyzed using a Bioanalyzer 2100 (*Agilent Technologies*). For sequencing, libraries were denatured with NaOH at 0.1 N and sequenced at 12 pmolar. Sample pools were distributed along the seven lanes of the flow cell, and one of the lanes was spared for sequencing of the control using PhiX (*Illumina, Inc*). Sequencing runs of 100 paired cycles (2 × 100) were performed with the *Hiseq 1500* platform*, Illumina.*

### RNA-Seq library preparation

Library for the RNA-Seq was performed using the *Truseq Stranded mRNA Sample Prep* KIT (*Illumina, Inc*)*,* according to the manufacturer's instruction. 1 µg of RNA was used for generating poly-A mRNA libraries from total RNA. Libraries were validated through quantification by real-time PCR using the *Kapa Library Quantification* kit *(Kapa Biosystems*)*,* and the size of the fragments was analyzed using a Bioanalyzer 2100 (*Agilent Technologies*) and the *High sensitivity DNA assay* kit (*Agilent Technologies*)*.* The concentration of all the samples was adjusted to 2 nM with Tris EDTA buffer. Sequencing of 100 paired cycles (2 × 100) was carried out in the *Hiseq 2500* platform using the fast mode.

### Bioinformatic analysis

FASTQ files for methylation analysis were processed using *fastQC*^68^ and Cutadapt (v. 2.10)^69^. Sequence alignment was run with Bismark v.0.19.1^70^ and Bowtie 2 (v. 2.2.5) against the reference genome GRCh38 with the specified options: -q -L 19 –score-min L,0,-0.2 -p 4 -reorder -ignore-quals -no-mixed -no-discordant -dovetail -maxins 500. The remaining parameters from Bismark were used as default. BAM files were subjected to the Bioconductor package Methylkit (v. 1.12.1)^71^, and bases in CpG context covered in all samples with depth above 10X and Phred quality score above Q20 were considered to carry out differential methylation analysis, hierarchical clustering, pairwise correlation coefficient, and Principal Component Analysis (PCA)^71^. Bases having more than 99.9^th^ percentile of coverage were excluded. To estimate sodium bisulfite conversion efficiency, the number of thymines was divided by total coverage for each non-CpG context. The percentage of methylation was calculated by dividing the number of cytosines by the total number of cytosines and thymines for each CpG context. Differential methylation analysis was performed creating non-overlapping tiling windows consisting of 100 bp, and the methylation percentage for a given region was compared against a control group. Methylkit employs logistic regression to detect Differentially Methylated Regions (DMR) and adjusts p-values to q-values with the Sliding Linear Model (SLIM) method^72^ to correct for multiple testing. Since a methylation difference greater than 25% was associated with a twofold repression in the gene expression^73^, we adopted a stringent moderate cutoff of ≥ 25% for methylation difference and a q-value < 0.01. GTF files containing genomic coordinates for DMR were annotated with Ensembl (v.92) to find DMR-associated genes. Biological pathway analysis for DMR was conducted through the WEB-based Gene SeT Analysis Toolkit^74^ using a database from the Kyoto Encyclopedia of Genes and Genomes (KEGG).R language^75^ (version 3.5.3) was utilized for statistical analysis. Hypo and hypermethylation difference among HIV groups were subjected to Kruskal–Wallis rank-sum test and Wilcoxon rank-sum test with Benjamini–Hochberg correction for multiple comparisons. Statistical significance for distances to TSS was calculated using the Wilcoxon rank-sum test. The cell fractions were estimated with the Bioconductor package EpiDISH version 2.10.0^76^. The robust partial correlation (RPC) inference was coupled with the reference dataset FlowSorted.Blood.450 k.compTable^77^ to estimate the cell fractions and the relative proportions for each cell subset were compared using the Kruskal–Wallis test.

The quality of the RNASeq libraries was evaluated using the fastQC^68^. We next used BWA-mem (v 0.7.17-r1188)^78^ for removal of contaminating ribosomal RNA (rRNA). The remaining reads were aligned to the reference genome GRCh37/hg19 using the STAR (v2.6.1a_08-27)^79^ aligner with paraments –chimSegmentMin 20, –limitIObufferSize 62500000 e-runThreadN 8. The resulting BAM files of accepted reads and GTF file with gene annotations (Ensembl v87) were used as inputs for HTSeq-count (v. 0.11.2)^80^ to obtain the gene-level counts. Using DEseq2 (v1.18.1)^81^, we assessed the differentially expressed genes among the samples. RNA-seq pathway analysis was performed according to the REACTOME database^82^. Network analysis was carried out with the Cytoscape platform^83^ coupled with GeneMANIA plugin^84^.

We then mapped DMR to differential expression genes and the Pearson correlation was made by using R scripts. Accession numbers pending. Raw data available on request.

### Ethics approval

The research was approved by the ethics committee from the Federal University of São Paulo (#51854).

## Supplementary Information

Supplementary Information 1.

Supplementary Information 2.

Supplementary Information 3.

Supplementary Information 4.

Supplementary Information 5.

Supplementary Information 6.

Supplementary Information 7.

Supplementary Information 8.

Supplementary Information 9.

## References

[1] Deaton AEM, Bird A (2011). CpG islands and the regulation of transcription. Genes Dev..

[2] Suzuki MM, Bird A (2008). DNA methylation landscapes: Provocative insights from epigenomics. Nat. Rev. Genet..

[3] Siliciano R, Greene W (2011). HIV latency. Cold Spring Harb. Perspect. Med..

[4] Kauder SE, Bosque A, Lindqvist A, Planelles V, Verdin E (2009). Epigenetic regulation of HIV-1 latency by cytosine methylation. PLoS Pathog.

[5] Blazkova J (2012). Paucity of HIV DNA methylation in latently infected, resting CD4+ T cells from infected individuals receiving antiretroviral therapy. J. Virol..

[6] Weber S (2014). Epigenetic analysis of HIV-1 proviral genomes from infected individuals: Predominance of unmethylated CpG's. Virology.

[7] Margolis DM (2011). Histone deacetylase inhibitors and HIV latency. Curr. Opin. HIV AIDS.

[8] Margolis DM, Garcia JV, Hazuda DJ, Haynes BF (2016). Latency reversal and viral clearance to cure HIV-1. Science.

[9] Siliciano JD (2003). Long-term follow-up studies confirm the stability of the latent reservoir for HIV-1 in resting CD4+ T cells. Nat. Med..

[10] Hermankova M (2001). HIV-1 drug resistance profiles in children and adults with viral load of< 50 copies/ml receiving combination therapy. JAMA.

[11] Archin NM (2009). Expression of latent human immunodeficiency type 1 is induced by novel and selective histone deacetylase inhibitors. AIDS.

[12] Samer S (2020). Nicotinamide activates latent HIV-1 ex vivo in ART suppressed individuals, revealing higher potency than the association of two methyltransferase inhibitors, chaetocin and BIX01294. Braz. J. Infect. Dis..

[13] Archin NM (2012). Administration of vorinostat disrupts HIV-1 latency in patients on antiretroviral therapy. Nature.

[14] Rasmussen TA (2014). Panobinostat, a histone deacetylase inhibitor, for latent-virus reactivation in HIV-infected patients on suppressive antiretroviral therapy: A phase 1/2, single group, clinical trial. Lancet HIV.

[15] Migueles SA (2009). Defective human immunodeficiency virus-specific CD8+ T-cell polyfunctionality, proliferation, and cytotoxicity are not restored by antiretroviral therapy. J. Virol..

[16] Shan L (2012). Stimulation of HIV-1-specific cytolytic T lymphocytes facilitates elimination of latent viral reservoir after virus reactivation. Immunity.

[17] Mikovits JA (1998). Infection with human immunodeficiency virus type 1 upregulates DNA methyltransferase, resulting in de novo methylation of the gamma interferon (IFN-γ) promoter and subsequent downregulation of IFN-γ production. Mol. Cell. Biol..

[18] Pion M, Jaramillo-Ruiz D, Martínez A, Muñoz-Fernández MA, Correa-Rocha R (2013). HIV infection of human regulatory T cells downregulates Foxp3 expression by increasing DNMT3b levels and DNA methylation in the FOXP3 gene. AIDS.

[19] Zhang Y (2015). Whole genome methylation array reveals the down-regulation of IGFBP6 and SATB2 by HIV-1. Sci. Rep..

[20] Straussman R (2009). Developmental programming of CpG island methylation profiles in the human genome. Nat. Struct. Mol. Biol..

[21] Zhang X (2016). Epigenome-wide differential DNA methylation between HIV-infected and uninfected individuals. Epigenetics.

[22] Meissner TB (2010). NLR family member NLRC5 is a transcriptional regulator of MHC class I genes. Proc. Natl. Acad. Sci..

[23] Oriol-Tordera B (2020). Methylation regulation of antiviral host factors, interferon stimulated genes (ISGs) and T-cell responses associated with natural HIV control. PLoS Pathog..

[24] Ke Q, Costa M (2006). Hypoxia-inducible factor-1 (HIF-1). Mol. Pharmacol..

[25] Semenza GL (2007). Hypoxia-inducible factor 1 (HIF-1) pathway. Sci. STKE.

[26] Dinarello CA (2011). A clinical perspective of IL-1β as the gatekeeper of inflammation. Eur. J. Immunol..

[27] Rose-John S (2018). Interleukin-6 family cytokines. Cold Spring Harb. Perspect. Biol..

[28] Zanoni M, Aventurato IK, Hunter J, Sucupira MCA, Diaz RS (2017). Uniquely altered transcripts are associated with immune preservation in HIV infection. PLoS ONE.

[29] Mohn F (2008). Lineage-specific polycomb targets and de novo DNA methylation define restriction and potential of neuronal progenitors. Mol. Cell.

[30] Payer B, Lee JT (2008). X chromosome dosage compensation: how mammals keep the balance. Annu. Rev. Genet..

[31] Stein R, Razin A, Cedar H (1982). In vitro methylation of the hamster adenine phosphoribosyltransferase gene inhibits its expression in mouse L cells. Proc. Natl. Acad. Sci..

[32] Wang GL, Jiang B-H, Rue EA, Semenza GL (1995). Hypoxia-inducible factor 1 is a basic-helix-loop-helix-PAS heterodimer regulated by cellular O2 tension. Proc. Natl. Acad. Sci..

[33] Phan AT, Goldrath AW (2015). Hypoxia-inducible factors regulate T cell metabolism and function. Mol. Immunol..

[34] Perlmann T, Wallén-Mackenzie Å (2004). Nurr1, an orphan nuclear receptor with essential functions in developing dopamine cells. Cell Tissue Res..

[35] Corley MJ (2016). Comparative DNA methylation profiling reveals an immunoepigenetic signature of HIV-related cognitive impairment. Sci. Rep..

[36] Kadkhodaei B (2009). Nurr1 is required for maintenance of maturing and adult midbrain dopamine neurons. J. Neurosci..

[37] Fang J-Y, Mikovits JA, Bagni R, Petrow-Sadowski CL, Ruscetti FW (2001). Infection of lymphoid cells by integration-defective human immunodeficiency virus type 1 increases de novo methylation. J. Virol..

[38] Youngblood B, Reich NO (2008). The early expressed HIV-1 genes regulate DNMT1 expression. Epigenetics.

[39] Nunes JM (2018). Modulation of epigenetic factors during the early stages of HIV-1 infection in CD4+ T cells in vitro. Virology.

[40] Jones PA (2012). Functions of DNA methylation: Islands, start sites, gene bodies and beyond. Nat. Rev. Genet..

[41] Saxonov S, Berg P, Brutlag DL (2006). A genome-wide analysis of CpG dinucleotides in the human genome distinguishes two distinct classes of promoters. Proc. Natl. Acad. Sci..

[42] Venter JC, Adams MD, Myers EW, Li PW, Mural RJ, Sutton GG, Smith HO, Yandell M, Evans CA, Holt RA, Gocayne JD, Amanatides P, Ballew RM, Huson DH, Wortman JR, Zhang Q, Kodira CD, Zheng XH, Chen L, Skupski M, Subramanian G, Thomas PD, Zhang J, Gabor Miklos GL, Nelson C, Broder S, Clark AG, Nadeau J, McKusick VA, Zinder N, Levine AJ, Roberts RJ, Simon M, Slayman C, Hunkapiller M, Bolanos R, Delcher A, Dew I, Fasulo D, Flanigan M, Florea L, Halpern A, Hannenhalli S, Kravitz S, Levy S, Mobarry C, Reinert K, Remington K, Abu-Threideh J, Beasley E, Biddick K, Bonazzi V, Brandon R, Cargill M, Chandramouliswaran I, Charlab R, Chaturvedi K, Deng Z, Di Francesco V, Dunn P, Eilbeck K, Evangelista C, Gabrielian AE, Gan W, Ge W, Gong F, Gu Z, Guan P, Heiman TJ, Higgins ME, Ji RR, Ke Z, Ketchum KA, Lai Z, Lei Y, Li Z, Li J, Liang Y, Lin X, Lu F, Merkulov GV, Milshina N, Moore HM, Naik AK, Narayan VA, Neelam B, Nusskern D, Rusch DB, Salzberg S, Shao W, Shue B, Sun J, Wang Z, Wang A, Wang X, Wang J, Wei M, Wides R, Xiao C, Yan C, Yao A, Ye J, Zhan M, Zhang W, Zhang H, Zhao Q, Zheng L, Zhong F, Zhong W, Zhu S, Zhao S, Gilbert D, Baumhueter S, Spier G, Carter C, Cravchik A, Woodage T, Ali F, An H, Awe A, Baldwin D, Baden H, Barnstead M, Barrow I, Beeson K, Busam D, Carver A, Center A, Cheng ML, Curry L, Danaher S, Davenport L, Desilets R, Dietz S, Dodson K, Doup L, Ferriera S, Garg N, Gluecksmann A, Hart B, Haynes J, Haynes C, Heiner C, Hladun S, Hostin D, Houck J, Howland T, Ibegwam C, Johnson J, Kalush F, Kline L, Koduru S, Love A, Mann F, May D, McCawley S, McIntosh T, McMullen I, Moy M, Moy L, Murphy B, Nelson K, Pfannkoch C, Pratts E, Puri V, Qureshi H, Reardon M, Rodriguez R, Rogers YH, Romblad D, Ruhfel B, Scott R, Sitter C, Smallwood M, Stewart E, Strong R, Suh E, Thomas R, Tint NN, Tse S, Vech C, Wang G, Wetter J, Williams S, Williams M, Windsor S, Winn-Deen E, Wolfe K, Zaveri J, Zaveri K, Abril JF, Guigo R, Campbell MJ, Sjolander KV, Karlak B, Kejariwal A, Mi H, Lazareva B, Hatton T, Narechania A, Diemer K, Muruganujan A, Guo N, Sato S, Bafna V, Istrail S, Lippert R, Schwartz R, Walenz B, Yooseph S, Allen D, Basu A, Baxendale J, Blick L, Caminha M, Carnes-Stine J, Caulk P, Chiang YH, Coyne M, Dahlke C, Mays A, Dombroski M, Donnelly M, Ely D, Esparham S, Fosler C, Gire H, Glanowski S, Glasser K, Glodek A, Gorokhov M, Graham K, Gropman B, Harris M, Heil J, Henderson S, Hoover J, Jennings D, Jordan C, Jordan J, Kasha J, Kagan L, Kraft C, Levitsky A, Lewis M, Liu X, Lopez J, Ma D, Majoros W, McDaniel J, Murphy S, Newman M, Nguyen T, Nguyen N, Nodell M, Pan S, Peck J, Peterson M, Rowe W, Sanders R, Scott J, Simpson M, Smith T, Sprague A, Stockwell T, Turner R, Venter E, Wang M, Wen M, Wu D, Wu M, Xia A, Zandieh A, Zhu X (2001). The sequence of the human genome. Science.

[43] Bird AP (1986). CpG-rich islands and the function of DNA methylation. Nature.

[44] Douek DC (2002). HIV preferentially infects HIV-specific CD4+ T cells. Nature.

[45] Brenchley JM (2004). T-cell subsets that harbor human immunodeficiency virus (HIV) in vivo: implications for HIV pathogenesis. J. Virol..

[46] Cohn LB (2015). HIV-1 integration landscape during latent and active infection. Cell.

[47] Elliott JH (2014). Activation of HIV transcription with short-course vorinostat in HIV-infected patients on suppressive antiretroviral therapy. PLoS Pathog..

[48] Fidler S (2020). Antiretroviral therapy alone versus antiretroviral therapy with a kick and kill approach, on measures of the HIV reservoir in participants with recent HIV infection (the RIVER trial): a phase 2, randomised trial. Lancet.

[49] Li E, Zhang Y (2014). DNA methylation in mammals. Cold Spring Harbor Perspect. Biol..

[50] Burnett JC (2010). Combinatorial latency reactivation for HIV-1 subtypes and variants. J. Virol..

[51] Scarpelini B (2016). Plasma metabolomics biosignature according to HIV stage of infection, pace of disease progression, viremia level and immunological response to treatment. PLoS ONE.

[52] Lee J-W, Bae S-H, Jeong J-W, Kim S-H, Kim K-W (2004). Hypoxia-inducible factor (HIF-1)α: Its protein stability and biological functions. Exp. Mol. Med..

[53] Zhuang X (2020). Hypoxic microenvironment shapes HIV-1 replication and latency. Commun. Biol..

[54] Deshmane SL (2009). Activation of the oxidative stress pathway by HIV-1 Vpr leads to induction of hypoxia-inducible factor 1α expression. J. Biol. Chem..

[55] Duette G (2018). Induction of HIF-1α by HIV-1 infection in CD4+ T cells promotes viral replication and drives extracellular vesicle-mediated inflammation. MBio.

[56] Mascanfroni ID (2015). Metabolic control of type 1 regulatory T cell differentiation by AHR and HIF1-α. Nat. Med..

[57] Chang C-H (2013). Posttranscriptional control of T cell effector function by aerobic glycolysis. Cell.

[58] Kang S, Tang H (2020). HIV-1 infection and glucose metabolism reprogramming of T cells: Another approach toward functional cure and reservoir eradication. Front. Immunol..

[59] Hegedus A, Williamson MK, Huthoff H (2014). HIV-1 pathogenicity and virion production are dependent on the metabolic phenotype of activated CD4+ T cells. Retrovirology.

[60] Palmer CS (2014). Glucose transporter 1-expressing Proinflammatory monocytes are elevated in combination antiretroviral therapy-treated and untreated HIV+ subjects. J. Immunol..

[61] Palmer CS (2014). Increased glucose metabolic activity is associated with CD4+ T-cell activation and depletion during chronic HIV infection. AIDS.

[62] Huang X, Liu G, Guo J, Su Z (2018). The PI3K/AKT pathway in obesity and type 2 diabetes. Int. J. Biol. Sci..

[63] Fang J, Zhou S-H, Fan J, Yan S-X (2015). Roles of glucose transporter-1 and the phosphatidylinositol 3-kinase/protein kinase B pathway in cancer radioresistance. Mol. Med. Rep..

[64] Bird A (2002). DNA methylation patterns and epigenetic memory. Genes Dev..

[65] Jaffe AE, Irizarry RA (2014). Accounting for cellular heterogeneity is critical in epigenome-wide association studies. Genome Biol.

[66] Jacoby M, Gohrbandt S, Clausse V, Brons NH, Muller CP (2012). Interindividual variability and co-regulation of DNA methylation differ among blood cell populations. Epigenetics.

[67] Horvath S (2013). DNA methylation age of human tissues and cell types. Genome Biol..

[68] ANDREWS, S. FastQC: A quality control tool for high throughput sequence data." *Reference Souce* (2010).

[69] Martin M (2011). Cutadapt removes adapter sequences from high-throughput sequencing reads. EMBnet. J..

[70] Krueger F, Andrews SR (2011). Bismark: a flexible aligner and methylation caller for Bisulfite-Seq applications. Bioinformatics.

[71] Akalin A (2012). methylKit: A comprehensive R package for the analysis of genome-wide DNA methylation profiles. Genome Biol..

[72] Wang H-Q, Tuominen LK, Tsai C-J (2011). SLIM: a sliding linear model for estimating the proportion of true null hypotheses in datasets with dependence structures. Bioinformatics.

[73] Avraham A (2014). Tissue specific DNA methylation in normal human breast epithelium and in breast cancer. PLoS ONE.

[74] Liao Y, Wang J, Jaehnig EJ, Shi Z, Zhang B (2019). WebGestalt 2019: gene set analysis toolkit with revamped UIs and APIs. Nucleic Acids Res..

[75] R, Team (2013). C.

[76] Teschendorff AE, Breeze CE, Zheng SC, Beck S (2017). A comparison of reference-based algorithms for correcting cell-type heterogeneity in epigenome-wide association studies. BMC Bioinform..

[77] Reinius LE (2012). Differential DNA methylation in purified human blood cells: implications for cell lineage and studies on disease susceptibility. PLoS ONE.

[78] Li H, Durbin R (2009). Fast and accurate short read alignment with Burrows–Wheeler transform. Bioinformatics.

[79] Dobin A (2013). STAR: Ultrafast universal RNA-seq aligner. Bioinformatics.

[80] Anders S, Pyl PT, Huber W (2015). HTSeq—a Python framework to work with high-throughput sequencing data. Bioinformatics.

[81] Love MI, Huber W, Anders S (2014). Moderated estimation of fold change and dispersion for RNA-seq data with DESeq2. Genome Biol..

[82] Fabregat A (2017). Reactome pathway analysis: A high-performance in-memory approach. BMC Bioinform..

[83] Otasek D, Morris JH, Bouças J, Pico AR, Demchak B (2019). Cytoscape automation: Empowering workflow-based network analysis. Genome Biol..

[84] Montojo J (2010). GeneMANIA Cytoscape plugin: fast gene function predictions on the desktop. Bioinformatics.

