# Supplementary material for: Latency-associated DNA methylation patterns among HIV-1 infected individuals with distinct disease progression courses or antiretroviral virologic response
Source: Sci Rep. 2021 Nov 26;11:22993. doi: 10.1038/s41598-021-02463-0 (PMC8626465; doi:10.1038/s41598-021-02463-0)
Supplement: Supplementary file 1 — Supplementary Information 1. [file 41598_2021_2463_MOESM1_ESM.docx]

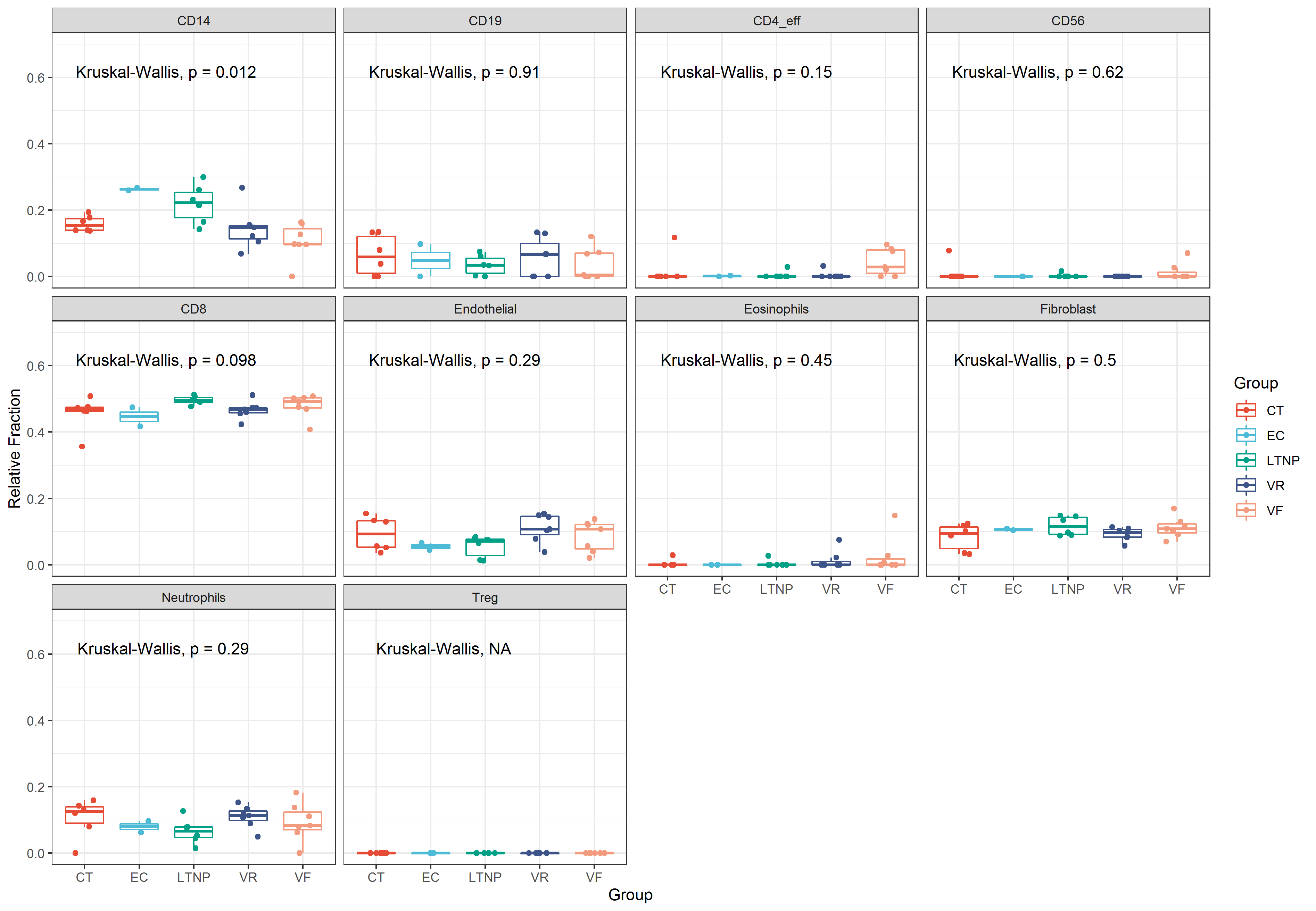


**Supplementary Figure 1: Deconvolution analysis.**

An *in silico* cell purification was performed in order to infer the cell type composition in the methylation data for each subject. The relative fractions for each cell subset were compared using the Kruskal-Wallis test.


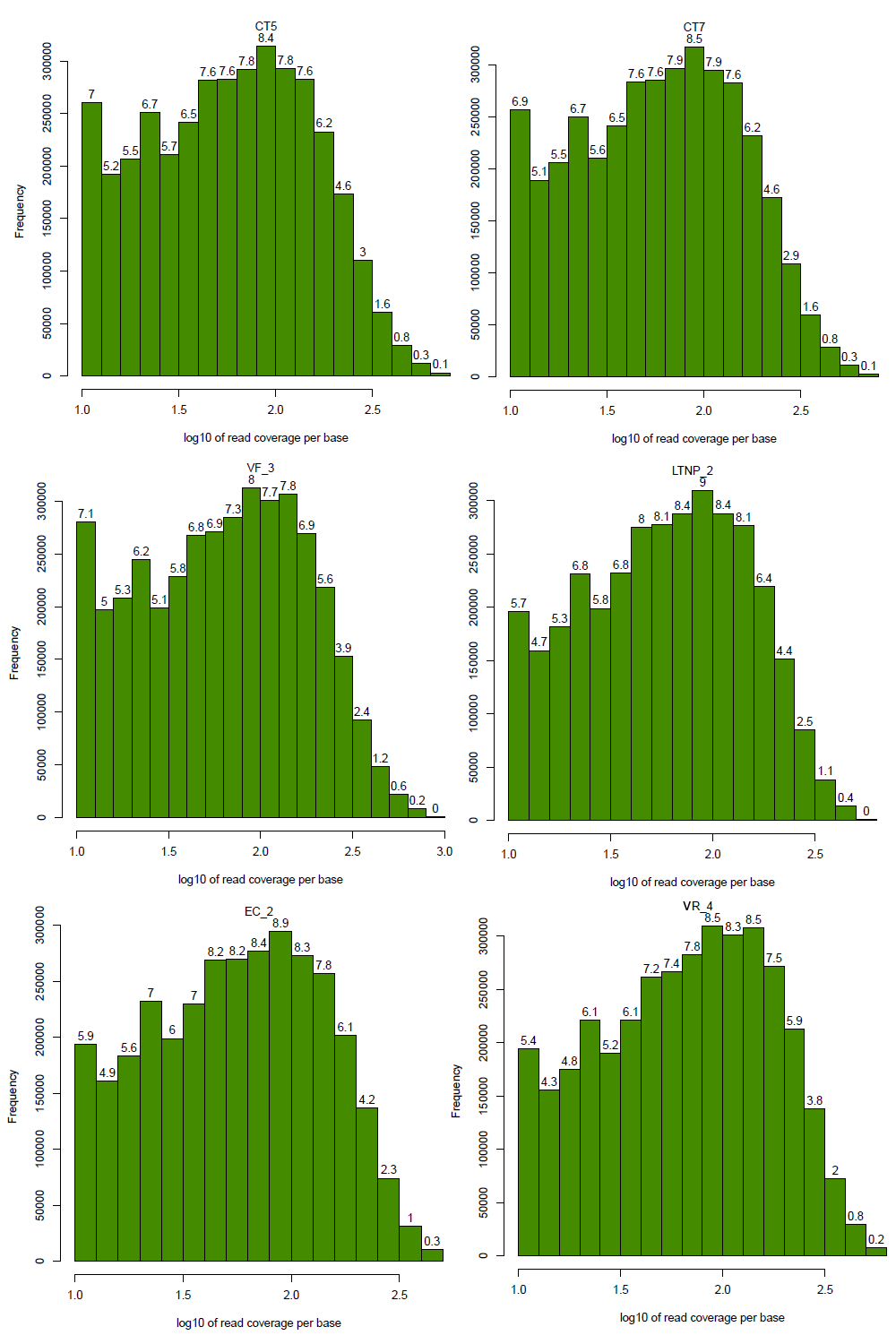


**Supplementary Figure 2: Distribution of sequencing coverage depth.**

CpG sequencing coverage depth of six samples representing the number of times each CpG was sequenced. Sequencing was performed as described in Methods.


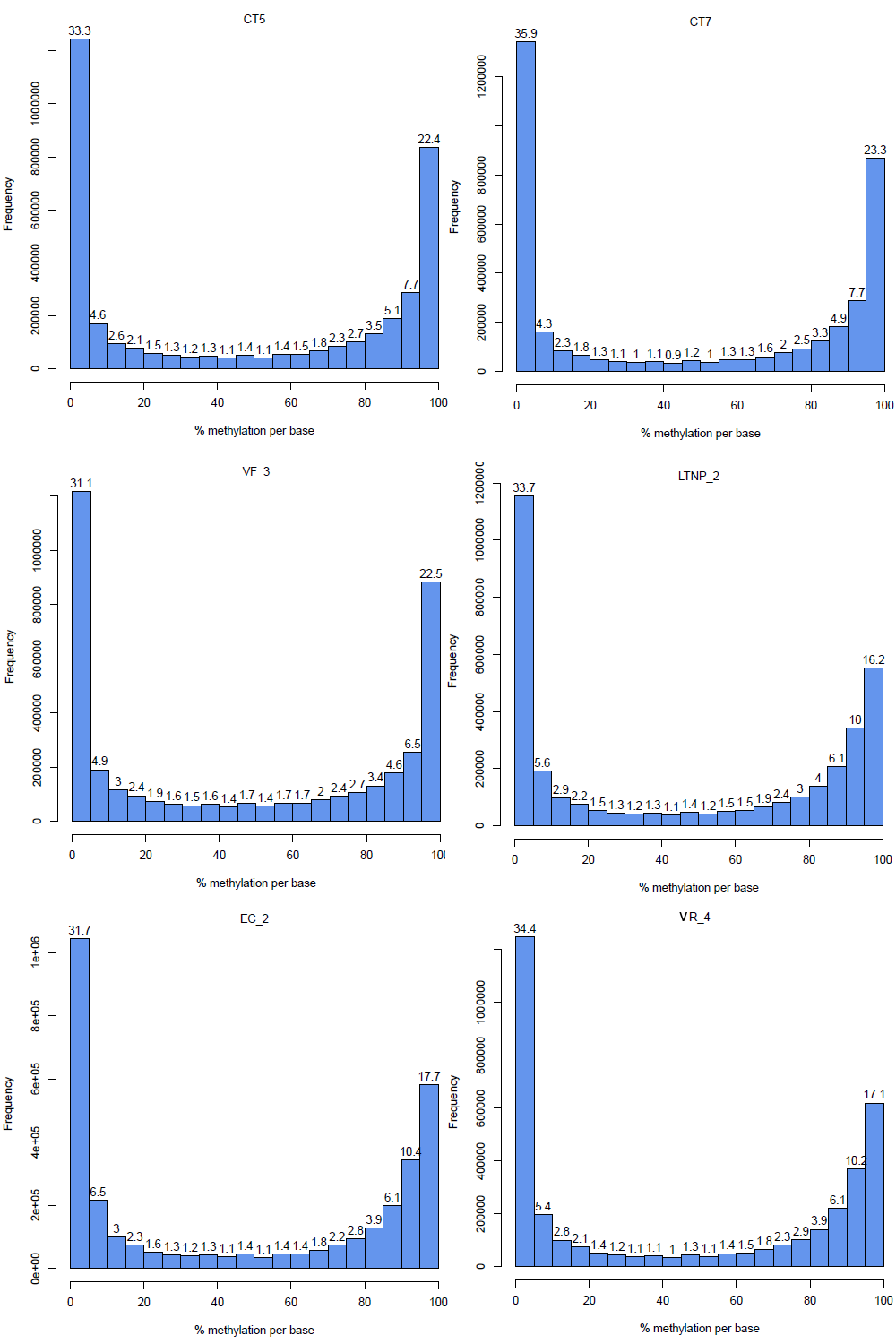


**Supplementary Figure 3: Methylation level distribution per base**.

Bimodal pattern of the methylation percentage distribution per CpG representing two main peaks of CpGs either unmethylated or fully methylated. Six out of 28 samples are represented. The percentage of methylation was calculated by dividing the number of cytosines by the total number of cytosines and thymines for each CpG context.


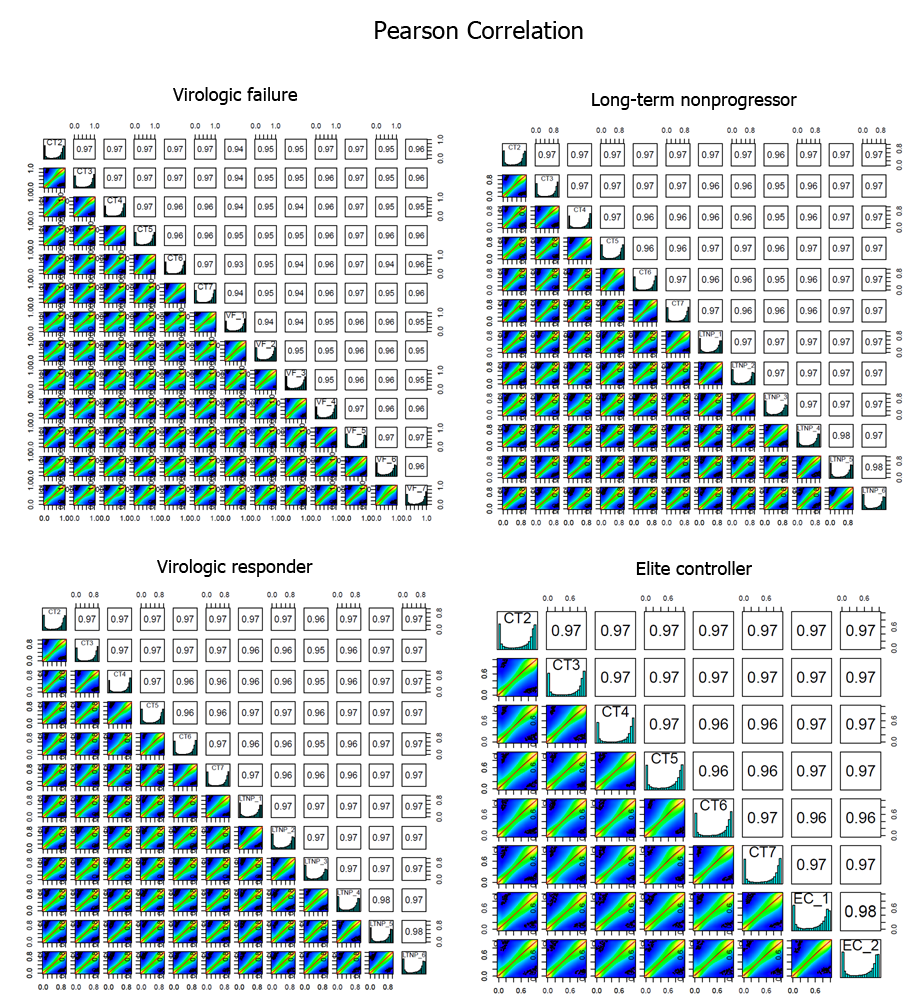


**Supplementary figure 4: Pearson correlation.**

Pair-wise Pearson correlation scores of methylation percentages in CpG regions are showed for each comparison between HIV infected groups and control.


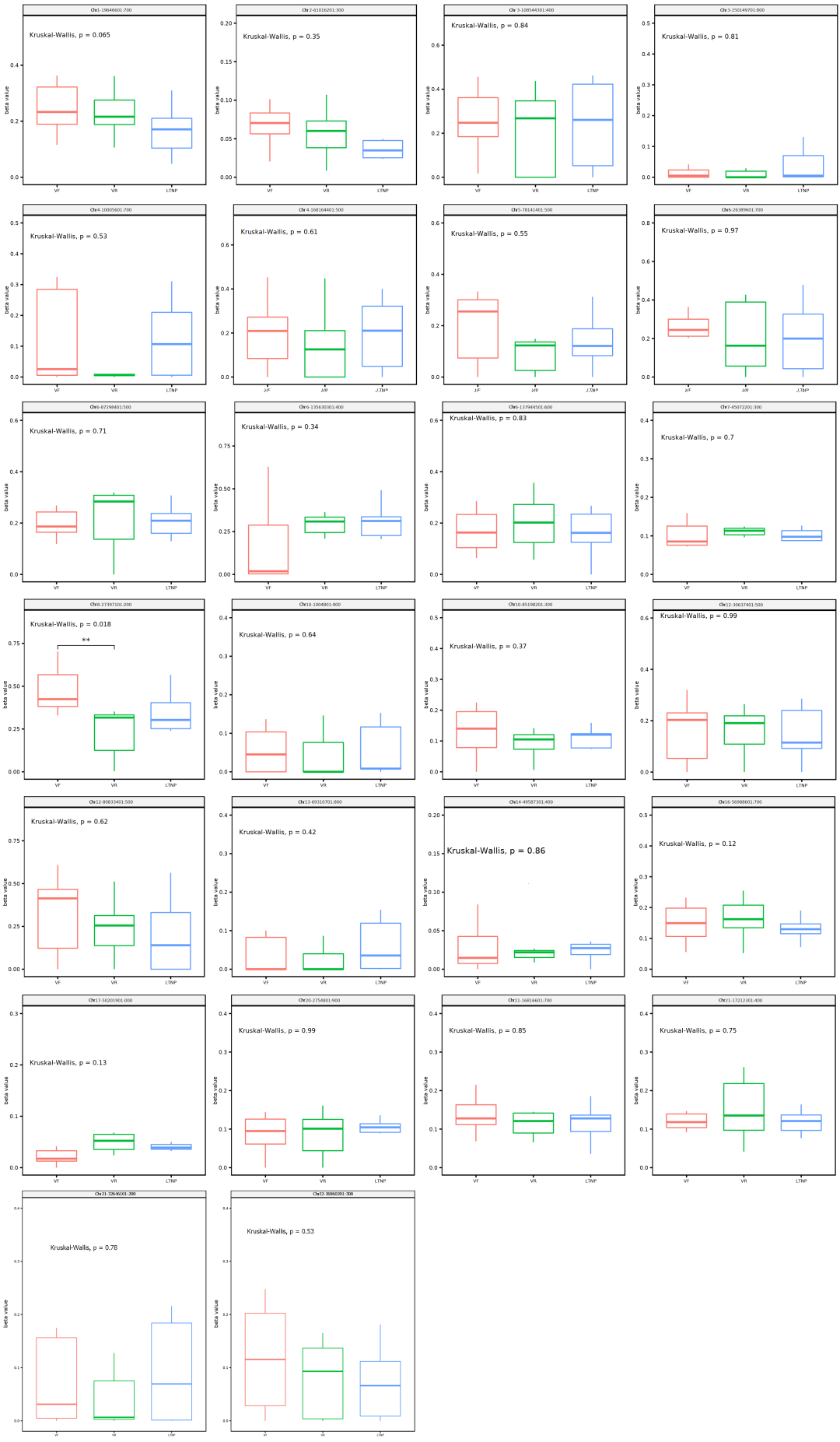


**Supplementary figure 5:**

Differentially hypomethylated regions in gene promoters of LTNP, virologic failures and responders were compared to address the significance of methylation changes for the individual groups.


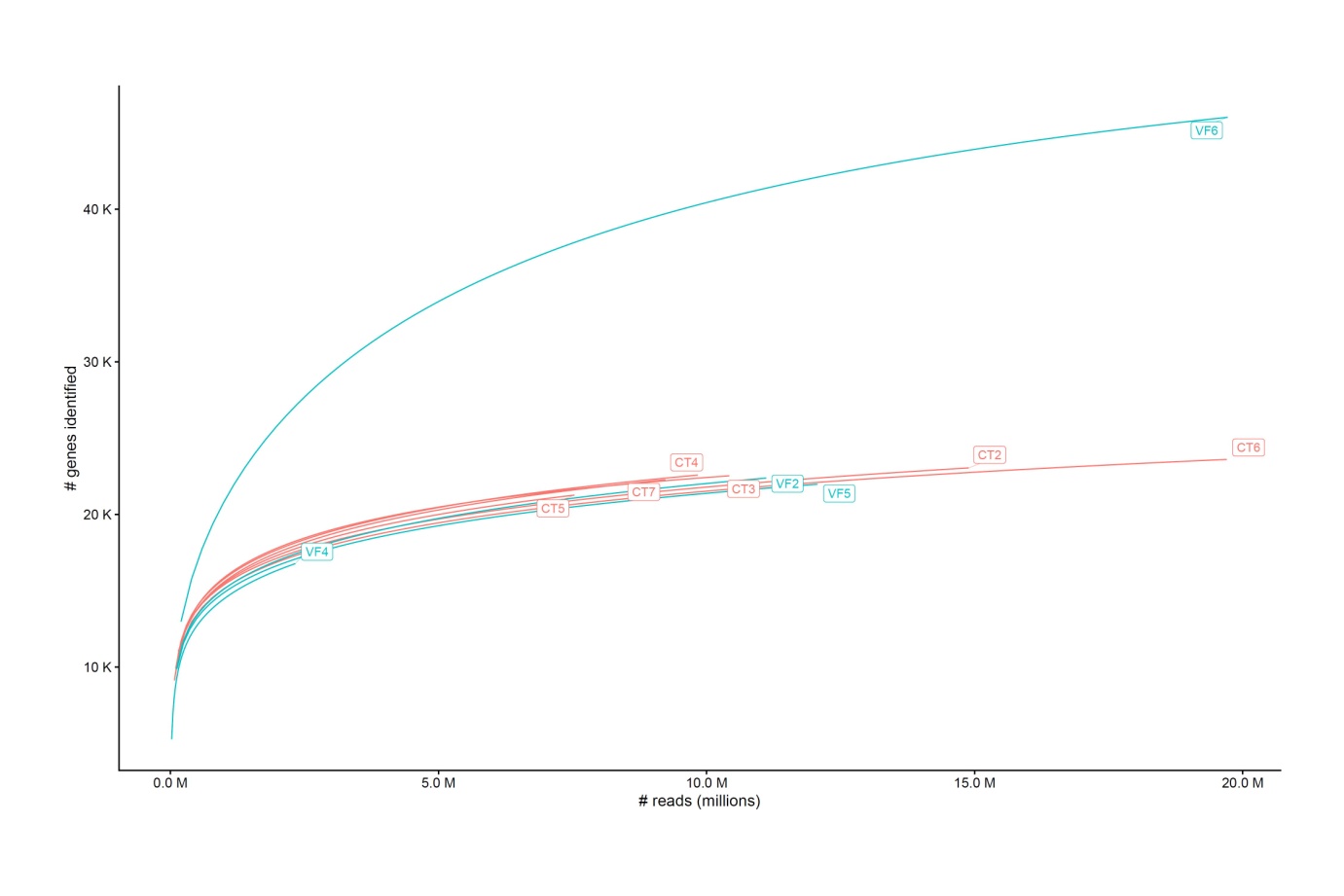


**Supplementary figure 6: Saturation curve for the RNA-seq analysis.**

Saturation curve denotes the number of reads sequenced in the x-axis and the number of transcripts identified in the y-axis for each sample.
