# Supplementary material for: Latency-associated DNA methylation patterns among HIV-1 infected individuals with distinct disease progression courses or antiretroviral virologic response
Source: Sci Rep. 2021 Nov 26;11:22993. doi: 10.1038/s41598-021-02463-0 (PMC8626465; doi:10.1038/s41598-021-02463-0)
Supplement: Supplementary file 9 — Supplementary Information 9. [file 41598_2021_2463_MOESM9_ESM.docx]

**Supplementary Tables**

**Supplementary Table 1**

**Class I HLA alleles**.

The HLA Class I alleles are depicted for the elite controllers and for four long term non-progressors.

**Supplementary Table 2**

**Fractions for Each Cell Subset in PBMC.**

The cell type abundance for the methylation data was estimated *in silico*. The relative fractions of each cell subset is shown.

**Supplementary Table 3**

**Final Alignment Report.**

The alignment report depicts the information regarding the sequencing output and bisulfate conversion rates for each sample.

**Supplementary Table 4**

**Differentially Methylated Regions.**

The methylation profiles of HIV groups were compared to control groups and are depicted regarding their location on the human genome, distance to TSS, methylation difference, p-value, and q-value.

**Supplementary Table 5**

**Biologic Pathways for DMR.**

The most significant DMR-associated biologic pathways are represented for hypo and hypermethylated regions.

**Supplementary Table 6**

**List of Differentially Expressed Genes.**

Gene expression of virologic failures was compared against control group. The complete list of DEG is provided considering log2 fold change > 3 and p-adjusted < 0.05.

**Supplementary Table 7**

**Biologic pathways for the RNA-seq data.**

The most significant biological pathways for DEG are shown.
